# The HER2 S310F Mutant Can Form an Active Heterodimer with the EGFR, Which Can Be Inhibited by Cetuximab but Not by Trastuzumab as well as Pertuzumab

**DOI:** 10.3390/biom9100629

**Published:** 2019-10-19

**Authors:** Jung Won Shin, Soohyun Kim, Suji Ha, Byungsan Choi, Seongyeong Kim, Seock-Ah Im, Tae-Young Yoon, Junho Chung

**Affiliations:** 1 Department of Biochemistry and Molecular Biology, Seoul National University College of Medicine, Seoul National University, Seoul 03080, Korea; yuanyang@snu.ac.kr; 2 Cancer Research Institute, Seoul National University College of Medicine, Seoul National University, Seoul 03080, Korea; kimchii481@gmail.com (S.K.); tnwlgk123@gmail.com (S.H.); seon86@snu.ac.kr (S.K.); moisa@snu.ac.kr (S.-A.I.); 3 Department of Cancer Biology, Seoul National University College of Medicine, Seoul 03080, Korea; 4 Department of Physics, Korea Advanced Institute of Science and Technology (KAIST), Daejeon 34141, Korea; cbs091004@gmail.com; 5 Biomedical Research Institute, Seoul National University Hospital, Seoul 03080, Korea; 6 Department of Internal Medicine, Seoul National University College of Medicine, Seoul 03080, Korea; 7 Department of Biological Sciences, Seoul National University, Seoul 08826, Korea; tyyoon@snu.ac.kr

**Keywords:** HER2, Mutation, Pertuzumab, TIR microscope, EGFR, Heterodimerization

## Abstract

G309 or S310 mutations on the HER2 extracellular domain II induce receptor activation. Clinically, S310F is most frequent among HER2 extracellular domain mutations and patients with the S310F mutation without HER2 amplification responded to trastuzumab with or without the pertuzumab combination. However, the ability of S310F mutant to form homodimers or heterodimers with wild-type HER2 and other HER receptors, or their reactivity to trastuzumab and pertuzumab treatments, has not been reported. We overexpressed S310F as well as G309A, G309E and S310Y HER2 mutants and tested their reactivity to trastuzumab and pertuzumab. All mutants reacted to trastuzumab, but S310F mutant did not react to pertuzumab along with S310Y or G309E mutants. Thereafter, we tested the effects of trastuzumab and pertuzumab on 5637 cell line expressing both wild-type HER2 and S310F mutant. The ligand-independent HER2 homodimerization blocking antibody, trastuzumab, did not inhibit the activation of the HER2 receptor, suggesting that the S310F HER2 mutant did not form homodimers or heterodimers with wild-type HER2. Because 5637 cells overexpressed the EGFR, the effects of cetuximab and gefitinib were determined, and both inhibited the activation of HER2 and significantly reduced cell growth. Because pertuzumab did not inhibit the phosphorylation of HER2 while it bound to wild-type HER2, EGFR-mediated phosphorylation is expected to occur on the S310F mutant. To confirm whether the S310F mutant HER2 retained its affinity to the EGFR, single molecule interaction analyses using TIRF microscopy were performed, which showed that S310F mutant successfully formed complexes with EGFR. In conclusion, HER2 S310F mutant can form an active heterodimer with the EGFR and it can be inhibited by cetuximab, but not by trastuzumab in combination with pertuzumab.

## 1. Introduction

HER2/ErbB2/Neu is a member of the human epidermal growth factor receptor (EGFR, HER) family of homologous transmembrane receptor tyrosine kinases. The HER family is composed of four receptors (EGFR and HER2–4/ErbB1–4) and a number of variants generated by alternative slicing. There are 11 types of ligands reactive to HER family receptors, including those generated by alternative splicing [1]. The roles for these receptors and their ligands have been well described in many types of human cancers, including breast, colon, pancreatic, ovarian, brain, and lung cancers [2]. Binding of the ligand to EGFR or HER3/4 induces conformational changes in the proteins, facilitating receptor dimerization, which results in transphosphorylation of tyrosine residues in the carboxy tail [3]. The phosphotyrosines are docking sites for the recruitment of downstream signaling proteins. Human epidermal growth factor receptor 2 (HER2) has no known ligand but forms heterodimers with other EGFR family members and activates downstream signaling through the phosphoinositide 3-kinase/AKT and MAPK pathways [4].

Amplification/overexpression of HER2 is associated with cell transformation and oncogenesis [5], is observed in 20–25% of breast cancers, and is associated with poor survival [6]. In breast cancer cells with HER2 gene amplification, HER2 receptors exist on the cell surface as monomers, homodimers and heterodimers. The therapeutic antibody trastuzumab, binding to extracellular domain IV, cannot block ligand-induced HER2 heterodimers and has preferential activity against breast cancers driven by HER2 homodimers [7,8]. Meanwhile, pertuzumab binds to extracellular domain II, sterically blocks a binding pocket necessary for receptor dimerization, and inhibits HER2 association with its partner receptors [9]. This property may explain why pertuzumab, unlike trastuzumab, is effective against a broad range of cancers which do not express HER2 at high levels [10,11]. Trastuzumab and pertuzumab could colocalize to HER2 while not augmenting the binding of each other [12]. Moreover, the combination of these two antibodies achieved significant improvement in the survival of HER2 overexpressing breast cancer patients. Antibody drug conjugates (trastuzumab emtansine), and small molecule tyrosine kinase inhibitors (lapatinib, erlotinib, and neratinib) have also dramatically improved the outcomes of HER2-positive cancer patients [13,14,15,16]. Besides the amplification/overexpression of HER2, somatic HER2 gene mutations have been detected in a range of human cancer types, including prostate neuroendocrine cancer, metastatic cutaneous squamous cell carcinoma, and bladder cancer [17,18]. Preclinical data suggest that functionally activating HER2 mutations may drive and maintain cancers in a manner analogous to HER2 gene amplification, and those HER2 mutations may similarly confer changes in sensitivity to HER2-directed drugs [19].

There are more than 20 extracellular domain mutations detected in human cancer tissues (Table 1) [8,19,20,21,22,23,24,25,26,27,28,29,30,31,32,33,34,35,36,37,38]. Furthermore, 26 additional extracellular domain mutations have been detected in various human cancer cell lines (Table 2). G309 and S310 mutations of HER2 domain II are well-known activating mutations. Previous studies showed that the G309 mutation leaves unpaired cysteine residues (C299–C311), forming reduction-sensitive dimers [20]. Likewise, the S310 mutation promotes noncovalent dimerization between neighboring molecules, promoting hyperphosphorylation of HER2 [20]. The Ba/F3 cells transfected with an expression vector encoding the S310F HER2 mutant showed hyperphosphorylated HER2 while cells overexpressing G309E mutant did not. However, Ba/F3 cells expressing either G309E or S310F HER2 mutant showed enhanced proliferation compared to cells transfected with wild-type HER2 and were sensitive to trastuzumab, lapatinib, afatinib, and neratinib [20]. Clinically, S310F is the most frequently found mutation in the HER2 extracellular domain [39]. Two HER2-nonamplified breast cancer patients with S310F mutation were successfully treated with the trastuzumab alone or in combination with pertuzumab [19,31]. G309 and S310 residues are located near the binding epitope for pertuzumab, obscuring the reactivity of the antibody [9]. However, the reactivity of the combination of pertuzumab and trastuzumab to the S310F mutant has not been previously reported. In this study, we prepared recombinant proteins corresponding to all reported G309 and S310 HER2 mutants and analyzed their interactions in Enzyme-linked immunosorbent assay (ELISA) with both trastuzumab and pertuzumab. We also used 5637 cells, an EGFR-amplified bladder cancer cell line expressing both S310F HER2 mutant and wild-type HER2 to analyze the downstream signaling of HER2 mutant and found that the S310F HER2 mutant efficiently formed heterodimers with EGFR, which could not be inhibited by the combination of trastuzumab and pertuzumab. And using single molecule level total internal reflection (TIR) microscopy, we confirmed that S310 mutant can form a heterodimer with EGFR at the molecular level.

## 2. Materials and Methods

### 2.1. Expression and Purification of Recombinant Fusion Proteins

Genes encoding the extracellular domain of wild-type HER2, wild-type HER3 and G309A, G309E, S310F, or S310Y HER2 mutants were chemically synthesized with *Sfi*I restriction sites at the 5′ and 3′ ends (GenScript Biotech, Jiangsu, China). After restriction digestion with *Sfi*I (New England Biolabs, Hertfordshire, UK), the genes were ligated into pCEP4 vectors encoding either human Fc or Cκ, as described previously [40,41]. Genes encoding the scFv form of pertuzumab, trastuzumab, and cetuximab were also synthesized with *Sfi*I restriction sites at the 5′ and 3′ ends (GenScript Biotech) and cloned into the human Cκ vector [42,43,44].

The expression vectors were then transfected into HEK293F cells (FreeStyle 293-F cells) using polyethylenimine (Sigma-Aldrich, St. Louis, MO, USA) as described previously [45]. The transfected cells were grown in FreeStyle 293 expression media (Invitrogen, Carlsbad, CA, USA) as described previously [46]. The culture supernatants were purified by affinity chromatography using either Protein A or KappaSelect resin (GE Healthcare, Buckinghamshire, UK) following the manufacturer’s instructions.

The purified recombinant proteins were subjected to 4%–12% NuPage bis-Tris gels (Invitrogen) according to the manufacturer’s instructions. After the electrophoresis, the gel was stained with Coomassie Brilliant Blue R-250 (Ameresco, Framingham, MA, USA).

### 2.2. ELISA

Recombinant wild-type HER2, wild-type HER3 or HER2 mutant extracellular domain-human Fc fusion proteins were coated overnight at 4 °C on the wells of microtiter plates (Corning, Corning, NY, USA). The wells were blocked with 130 μL of blocking buffer (3% bovine serum albumin (BSA) in phosphate-buffered saline (PBS)) for 1 h at 37 °C. The plates were then incubated with recombinant pertuzumab or trastuzumab scFv- human Cκ fusion proteins serially diluted four-fold in blocking buffer for 2 h at 37 °C. The plates were then washed with 150 μL of 0.05% Tween 20 in PBS (PBST) for three times. Subsequently, horseradish peroxidase (HRP) conjugated anti-human Cκ light chain antibody (1:5000; Millipore, Hayward, CA, USA) was added to each well. After incubation for 1 h at 37 °C, the plates were washed. Finally, 50 μL of 3,3′,5,5′-tetramethyl benzidine substrate solution (TMB) substrate was added and optical density was measured at 650 nm (LabSystems Diagnostics Oy, Vantaa, Finland).

### 2.3. Cell Culture

The 5637and AU565 cells were obtained from the Korean Cell Line Bank (Seoul, Republic of Korea). The cells were grown in RPMI-1640 media (Welgene, Seoul, Republic of Korea) supplemented with 10% fetal bovine serum (Thermo Fisher Scientific, Gibco, Waltham, MA, USA) and 1% penicillin-streptomycin (1000 U/mL) (Thermo Fisher Scientific, Gibco).

### 2.4. Sequencing of Amplified HER2 Gene Fragments of 5637 Cell

Total RNA was isolated from the 5637 cells using TRIzol reagent (Invitrogen) as previously described [47]. After cDNA was synthesized using a SuperScript III First-Strand Synthesis system (Invitrogen), the HER2 gene fragment encoding from N302 residue to R340 residue was amplified using specific primer sets (HER2 forward: 5′-GCCTCCACTTCAACCACAGTGGC-3′ and HER2 reverse: 5′-CTGTGATCTCTTCCAGAGTCTCAAAC-3′). The PCR conditions were as follows: Preliminary denaturation at 95 °C for 7 min, followed by 25 cycles of 30 s at 95 °C, 30 s at 54 °C, and 1 min at 72 °C. The reaction was ended with an extension step for 5 min at 72 °C. After agarose gel electrophoresis, the amplified DNA was extracted using the Qiagen Gel Extraction Kit according to the manufacturer’s instructions (Qiagen, Hilden, Germany) and subjected to Sanger sequencing (Macrogen, Seoul, Korea).

### 2.5. Flow Cytometry Analysis

The 5637 and AU565 cells (3 × 10^5^ cells/well, Corning) were resuspended in 100 µL of flow cytometry buffer (1% BSA and 0.02% sodium azide in PBS), then incubated with cetuximab, pertuzumab, or trastuzumab scFv- human Cκ fusion proteins at a final concentration of 1 μM for 1 h at 37 °C. The cells were then washed twice with 1% (*w*/*v*) BSA in PBS and incubated with FITC-conjugated anti-human Cκ light chain antibody (Thermo Fisher Scientific) for 40 min at 37 °C. After washing with flow cytometry buffer, the fluorescence intensity of cells was measured using a FACS Canto II (BD Bioscience, Heidelberg, Germany) and analyzed with FlowJo data analysis software, version 8.8.4 (Threestar, OR, USA). Non-transfected HEK293T cell and cells transfected with EGFR-mCherry and HER2-eGFP bicistronic expression vector were used as control cells and were analyzed in the same manner as above.

### 2.6. Immunoprecipitation and Immunoblot Analyses

The AU565 and 5637 cells (1 × 10^7^ cells) were lysed in 100 μL of cold RIPA buffer (150 mM NaCl, 1% Triton X-100, 1% sodium deoxycholate, 0.1% sodium dodecyl sulfate, 50 mM Tris-HCl, and 2 mM EDTA at pH 7.5) containing a protease inhibitor cocktail (Roche, Basel, Switzerland). Subsequently, 500 μL of the cell lysate was incubated with either recombinant trastuzumab or pertuzumab scFv-human Cκ fusion protein with a final concentration of 100 μg/mL at 4 °C overnight with rotation. Then, 10 μL of KappaSelect resin (GE Healthcare) was added to the lysate, followed by incubation at 4°C for 4 h with gentle rotations. After centrifugation for 3 min at 1200× *g*, the resin and supernatant were collected. The cell lysate, resin, and supernatant representing the same number of the cells were subjected to SDS-polyacrylamide gel electrophoresis (SDS-PAGE) as described above and transferred onto nitrocellulose membranes (Whatman, Dassel, Germany) using a transfer system as previously described [48]. Thereafter, the membrane was blocked with 5% skim milk (BD Biosciences, CA, USA) followed by incubation with rabbit anti-HER2 antibody (Cell Signaling Technology, Danvers, MA, USA) at 4 °C overnight and with horseradish peroxidase (HRP)-conjugated goat anti-rabbit IgG (1:5000, 111-035-008; Jackson Immuno Research Laboratory, West Grove, PA, USA). After several washes with Tris-buffered saline and Tween [10 mM Tris-HCl, pH 7.5, 150 mM NaCl, and 1% (*v*/*v*) Tween 20], the protein bands were visualized using Super Signal Pico West chemiluminescent substrate (#34080; Thermo Fisher Scientific) and a gel doc EZ system (Bio-Rad, Hercules, CA, USA).

### 2.7. Cell Viability Assay and Immunoblotting

The 5637 cells were seeded at a density of 5.5–10 × 10^3^/well in 96-well culture plates. After incubation for 24 h, cells were treated with 1 μM of cetuximab (Merck, Palo Alto, CA, USA), pertuzumab (Roche), trastuzumab (Roche), gefitinib (AstraZeneca, Cambridge, UK), or lapatinib (GlaxoSmithKline, Brentford, UK). After 96 h, the number of viable cells was determined using a Premix WST-1 kit (Takara, Kyoto, Japan) following the manufacturer’s protocol. For immunoblot analysis, 5637 cells were lysed in ice-cold RIPA buffer containing a protease inhibitor cocktail (Roche) and phosphatase inhibitor (Roche). The cell lysates were cleared by centrifugation for 10 min at 13,000× *g* and the amount of protein in the supernatants was determined by a BCA assay (Pierce Biotechnology, Waltham, MA, USA). The proteins in the supernatants were separated by SDS-PAGE using 4%–12% bis-Tris gels (Invitrogen) as described above and transferred onto a nitrocellulose membrane. The membrane was blocked by pre-incubation in 5% BSA/0.2% TBST at room temperature for 30 min and then incubated with anti-EGFR antibody (Cell Signaling Technology), anti-phospho-EGFR (Tyr 1068) antibody (Cell Signaling Technology), anti-HER2 antibody (Cell Signaling Technology), anti-phospho-HER2 (Tyr 1221/1222) antibody (Cell Signaling Technology), anti-PARP (Cell Signaling Technology), anti-cleaved PARP (Cell Signaling Technology), or anti-β-actin antibody (Cell Signaling Technology) for overnight at 4 °C. After washing three times with 0.2% TBST, the membrane was incubated with either HRP-conjugated goat anti-rabbit IgG Fc antibody (#31463; Thermo Fisher Scientific) or HRP-conjugated goat anti-mouse IgG antibody (sc-2005; Santa Cruz Biotechnology, Santa Cruz, CA, USA) for 1 h at room temperature. The membrane was washed three times with TBST, and the bound antibody was visualized by addition of Super Signal Pico West chemiluminescent substrate (#34080; Thermo Fisher Scientific) following the manufacturer’s instructions. The Image Lab program (BioRad, CA, USA) was used to determine the intensity of bands.

### 2.8. Single-Molecular Interaction Analysis Using Total Internal Reflection Fluorescence (TIRF) Microscopy

Single-molecular interaction analysis was performed as described in a previous study with appropriate modifications [49]. Genes encoding fusion proteins composed of the extracellular domain of the EGFR fused with mCherry protein (EGFR-mCherry) and that of wild-type HER2 fused with eGFP (HER2-eGFP) were ligated into a bicistronic expression vector. Another expression vector was prepared with the S310F mutant (S310F HER2-eGFP) replacing wild-type HER2.Then, these vectors were transfected into HEK293T cells (FreeStyle 293-T cells). After culture at 37 °Cwith 5% CO_2_ for 1 day, the cells were dissolved in lysis buffer (1% Triton X-100, 150 mM NaCl, 1 mM EDTA, 10% glycerol) with protease (P8340; Sigma-Aldrich) and phosphatase (P5726; Sigma-Aldrich) inhibitor cocktail. After centrifugation at 15,000× *g* for 10 min at 4 °C, the supernatant was collected. Recombinant mCherry (4999-100; Biovision, Milpitas, CA, USA) and eGFP (4993-100; Biovision) protein standards were used to determine the concentration of fluorescently tagged proteins in the cell lysates. The fluorescence levels for recombinant eGFP and mCherry proteins of known concentrations (5–25 nM, five different concentration points) were measured using a fluorometer (Enspire 2300; Perkin-Elmer, San Jose, CA, USA) and were used to construct a calibration curve.

Detailed procedures of the flow chamber construction are described in previous studies [50,51]. The flow chamber was washed twice with 200 μL of PBS. Afterwards, 50 μL of NeutrAvidin solution (A2666; Invitrogen) was added to the flow chamber and was incubated for 5 min at room temperature. Subsequently, the flow chambers were washed twice followed by addition of diluted biotinylated polyclonal RFP antibody (34771; Abcam, Cambridge, UK) for 5 min at room temperature. This antibody was reported to be reactive to all RFP variants from *Discosoma,* including mCherry. After the flow chambers were washed again, cell lysates were added and incubated for 10 min at room temperature. Finally, the flow chambers were washed with 0.1% lysis buffer/PBS. EGFR/HER2 heterodimers were then detected with a TIRF microscope equipped with a 488-nm laser for eGFP detection. The images were recorded at 0.1 s per frame, and 20 frames were recorded. To obtain a time-averaged image, three frames were averaged. EGFP spots were characterized from these time-averaged images. To obtain the intensity of eGFP spots, 10 time-averaged images were used.

## 3. Results

### 3.1. The Recombinant S310F Mutant Is Not Reactive to Pertuzumab but Binds to Trastuzumab

**Figure 1:**
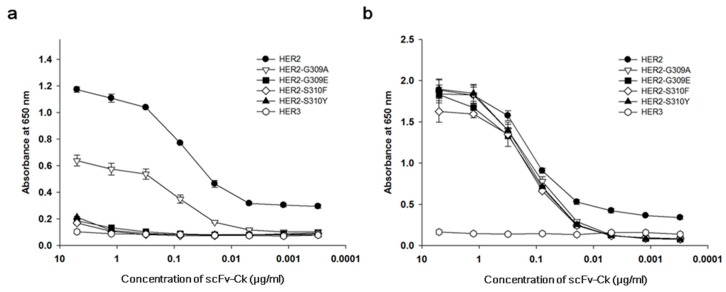
Reactivity of G309 and S310 HER2 mutants to pertuzumab and trastuzumab. Recombinant G309 and S310 mutant, wild-type HER2 or wild-type HER3 human Fc fusion protein was coated onto the wells of microtiter plates. The plate was blocked and subjected to incubation with (**a**) recombinant pertuzumab scFv-human Cκ fusion protein, or (**b**) trastuzumab scFv-human Cκ fusion protein at varying concentrations. Wild-type HER3 human Fc fusion protein was used as a negative control, because it does not bind to either antibody. The amount of bound antibody was determined using HRP-conjugated human Cκ light chain antibody and 3,3′,5,5′-tetramethyl benzidine (TMB) substrate solution.

A construct encoding the S310F HER2 extracellular domain fused to a human Fc domain of immunoglobulin heavy chain was prepared and cloned into a mammalian expression vector. For comparison, the expression vectors encoding the extracellular domains of all the other G309 and S310 mutants reported previously (G309A, G309E, S310Fand S310Y HER2 mutants) and those of wild-type HER2 and HER3 were also prepared. After transfection, the recombinant fusion proteins were purified from the culture supernatant using an affinity column reactive to human Fc. The extracellular domain of four mutants and wild-type HER2 was also prepared as a fusion protein of human Cκ through cloning into a mammalian expression vector, transfection and purification using an affinity column reactive to human Cκ from the culture supernatant. To check the purity, the recombinant fusion proteins were subjected to SDS-polyacrylamide gel electrophoresis and the gel was stained. No band except the fusion protein was visualized. In non-reducing conditions, multimers of G309E HER2 mutant fusion proteins were visualized in both fusion protein forms (Figure S1). The purified recombinant Fc fusion proteins were then subjected to an ELISA to test reactivity to pertuzumab and trastuzumab. A microtiter plate was coated with the recombinant Fc fusion proteins, blocked, and incubated with either pertuzumab or trastuzumab expressed as recombinant scFv- human Cκ fusion protein. Then, the amount of bound antibody was determined using anti-human Cκ antibody conjugated to HRP. S310F mutants did not bind to pertuzumab but reacted with trastuzumab in a dose-dependent manner (Figure 1). Pertuzumab did not bind to the G309E, S310F, and S310Y HER2 mutants, but reacted with the S309A HER2 mutant with reduced affinity compared to that of the wild-type HER2. Trastuzumab bound to the wild-type HER2 and all G309 and S310 HER2 mutants in a dose-dependent manner.

### 3.2. A Bladder Cancer Cell Line, 5637, Expresses Both Wild-Type HER2 and S310F Mutant

**Figure 2:**
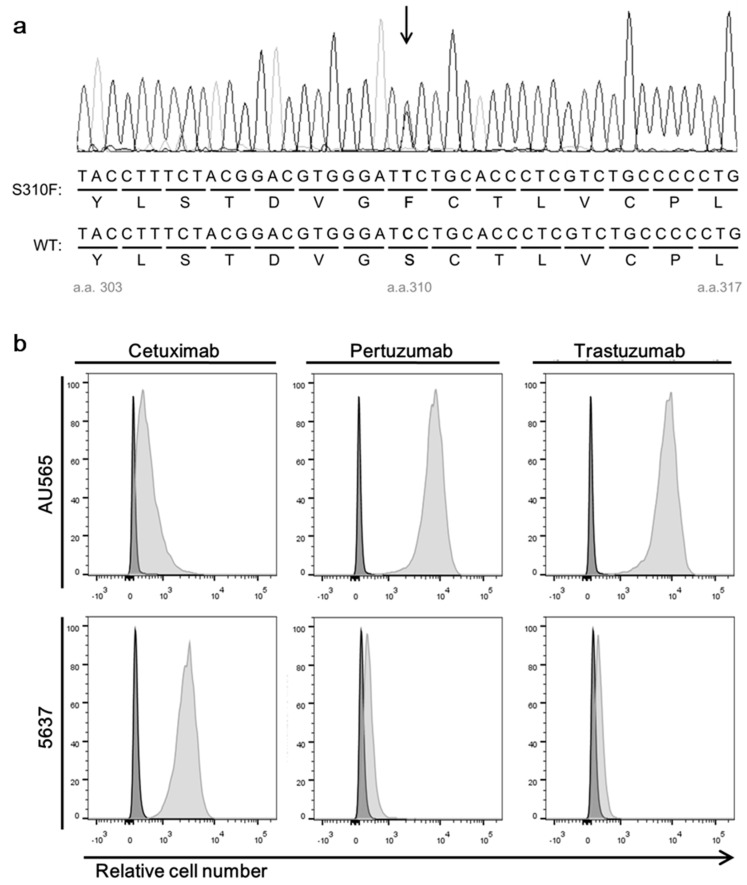
Expression of HER2 and epidermal growth factor receptor (EGFR) in 5637 and AU565 cells. (**a**) A representative sequence chromatogram showing the presence of two transcripts encoding wild-type HER2 and the S310F mutant in 5637 cells. (**b**) Flow cytometry analysis of two cancer cell lines assessing their reactivity to cetuximab, pertuzumab, and trastuzumab. The cells were incubated with individual antibody using the recombinant scFv-human Cκ fusion protein. The amount of bound antibody was determined using Allophycocyanin (APC)-labeled anti-human Cκ antibody.

We searched literatures to identify a human cell line expressing the S310F mutant and found that bladder cancer cell line 5637 expressed the mutant. To test the allelic expression of the S310F HER2 mutant in the 5637 cell line, we extracted total RNA from the cells and prepared cDNA. A HER2 gene fragment including the S310 residue was amplified by PCR using specific primers and subjected to Sanger sequencing. A representative sequence chromatogram is shown in Figure 2. We found that both the wild-type (nucleotide C) and the mutant (nucleotide T) peaks co-existed at the corresponding nucleotide position (Figure 2a). To check the expression of the wild-type and mutant HER2 on the cell surface, we used recombinant trastuzumab and pertuzumab scFv-Cκ fusion proteins to avoid the possible non-specific interaction through Fc regions of IgG and Fc receptors on the cell and performed flow cytometric analyses. As expected, the combination of pertuzumab and the trastuzumab scFv-Cκ fusion protein reacted with the 5637 cells (Figure 2b), which proved the presence of the wild-type HER2 on the cell surface of 5637 cells.

To confirm the expression of the S310F HER2 mutant in 5637 cells, we used immunoprecipitation experiments. We hypothesized that if the cells expressed the S310F HER2 mutant, it would not be immunoprecipitated by pertuzumab and would remain in the cell lysate. To determine the saturable amount of pertuzumab for immunoprecipitation experiments, we screened cell lines expressing higher amounts of HER2 than 5637 cells and identifiedAU565 cells (Figure 2b). The amount of pertuzumab and trastuzumab scFv-Cκ fusion protein guaranteeing the immunoprecipitation of all of HER2 in AU565 cell lysates was then determined (Figure S2). This predetermined amount of scFv-human Cκ fusion protein was then incubated with the 5637 cell lysate, with added affinity resin reactive to Cκ. After centrifugation, pellets and supernatants were collected. Thereafter, the cell lysate, precipitated resin, and supernatant representing the same number of 5637 cells were subjected to SDS-polyacrylamide gel electrophoresis and immunoblot analysis using anti-HER2 antibody reactive to the HER2 domain III. Trastuzumab scFv-Cκ fusion protein immunoprecipitated nearly all HER2 moleucules in the 5637 cell lysate, but pertuzumab scFv-human Cκ fusion protein immunoprecipitated only a fraction of HER2 (Figure S2). With this observation, we concluded that 5637 cells expressed significant amounts theS310F mutant.

### 3.3. EGFR Activates S310F HER2 Mutant in 5637 Cell

**Figure 3:**
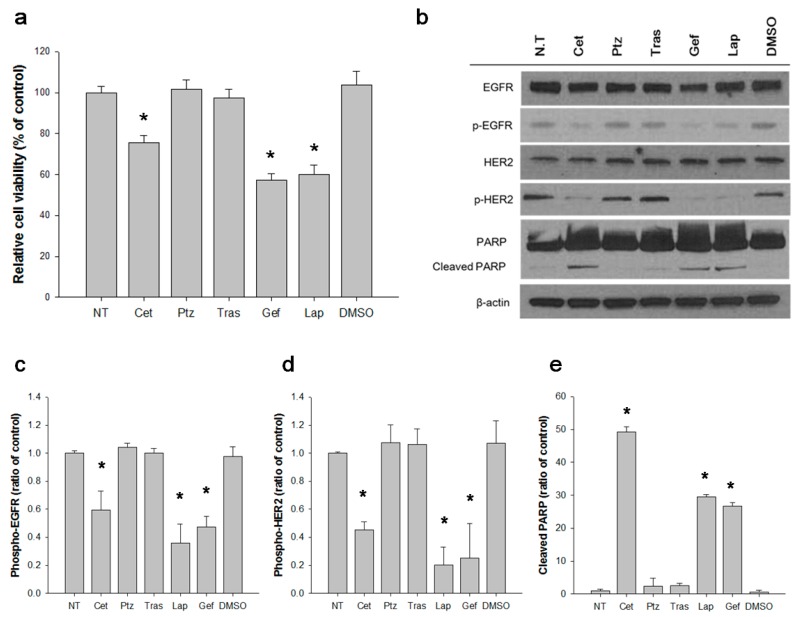
Inhibition of 5637 cell proliferation and receptor activation by anti-HER2 and anti-EGFR agents. Cells were treated with HER2 or EGFR inhibitors for 96 h. (**a**) Cell viability was measured by WST-1 kit as described in materials and methods. Relative cell viability as a percent was determined as ((absorbance in each treatment set—absorbance in untreated set)/absorbance in non-treated set X 100). Results represent the mean ± SD obtained from three independent experiments. * *p* < 0.05. (**b**) The cell lysate was subjected to immunoblot analysis to visualize the relative phosphorylation level EGFR (**c**) and HER2 (**d**) and the level of cleaved PARP (**e**) Each of these levels were quantified by densitometry and plotted, individually. * *p* < 0.005. NT; not treated, Cet; Cetuximab, Ptz; Pertuzumab, Gef; Gefitinib, Lap; Lapatinib, DMSO; dimethyl sulfoxide, PARP; poly (ADP-ribose) polymerase.

We tested the effects of anti-HER2 agents on 5637 cell proliferation and the level of HER2 phosphorylation at Y1221 and Y1222 residues. The cells were incubated with pertuzumab, trastuzumab and lapatinib for 96 h, lysed and subjected to SDS-PAGE and immunoblot analysis using anti-phospho-HER2 antibody. In parallel, the number of viable cells after the incubation was determined. Inhibiting the ligand-independent dimerization of wild-type and S310F HER2 mutant by trastuzumab did not reduce the cell proliferation or HER2 phosphorylation (Figure 3). Inhibiting the ligand-dependent dimerization of wild-type HER2 by pertuzumab also failed to show any effect (Figure 3). However, lapatinib inhibited the cell proliferation and reduced HER2 phosphorylation (Figure 3a,b,d). Because lapatinib inhibits the catalytic activity of both the EGFR and HER2, we then tested the effects of cetuximab and gefitinib, and both effectively inhibited the cell proliferation and reduced the levels of phosphorylated HER2and EGFR (Figure 3). The apoptosis induced by anti-EGFR and anti-HER2 agents was also determined (Figure 3b,d). In accordance with the cell proliferation data, only cetuximab, gefitinib, and lapatinib induced the cleavage of PARP (Figure 3b,e). Based on these data, we concluded that EGFR induced phosphorylation of S310F mutant in 5637 cells.

### 3.4. Single-Molecular Interaction Analysis Demonstrated That the S310F HER2 Mutant Formed Heterodimers with the EGFR

**Figure 4:**
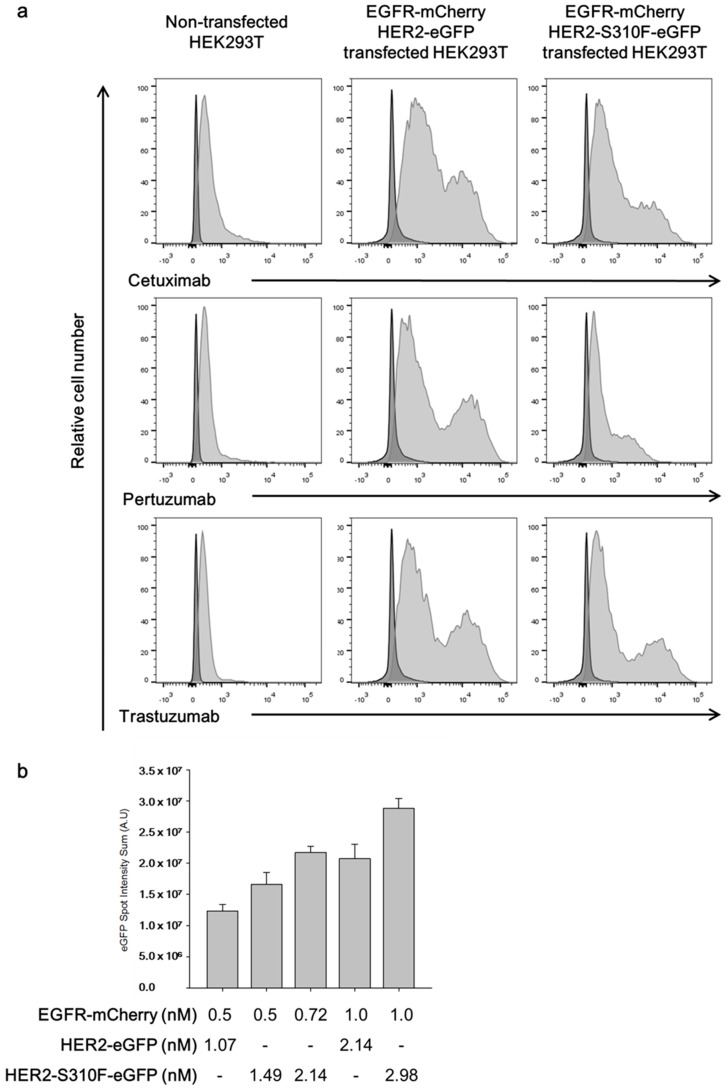
Single-molecular interaction analysis for the S310F mutant and EGFR heterodimerization. HEK293T cells were transfected with expression vectors encoding either EGFR-mCherry and wild-type HER2-eGFP or EGFR-mCherry and S310F HER2-eGFP fusion proteins. (**a**) After transfection, the cell surface expression of EGFR and HER2 or HER2 was checked by flow cytometry analysis using cetuximab, pertuzumab, and trastuzumab in the scFv-human Cκ format and APC-labeled anti-human Cκ antibody. (**b**) The lysates were subjected to a flow chamber coated with anti-mCherry antibody. Then, the summed intensity of eGFP spots was measured and plotted. Results represent the mean ± SD obtained from experiments performed in triplicate.

Single molecule interaction analysis was performed to determine whether the S310F HER mutant formed a heterodimer with the EGFR. We constructed a bicistronic mammalian expression vector encoding EGFR-mCherry and S310F HER2-eGFP fusion proteins. For comparison, we also prepared another expression vector encoding EGFR-mCherry and wild-type HER2-eGFP fusion proteins. After transfection of the expression vector into HEK293T cells, the expression of EGFR, S310F HER2 mutant, and wild-type HER2 were confirmed by flow cytometry analysis; using recombinant cetuximab, trastuzumab and pertuzumab scFv-human Cκ fusion proteins, the HEK293T cells showed basal expression of EGFR and HER2 (Figure 4a). The transfected cells showed significantly increased reactivity to cetuximab and trastuzumab. In HEK293T cells transfected with the expression vector encoding EGFR-mCherry and S310F HER2-eGFP, the reactivity with pertuzumab was significantly lower compared to the cells transfected with that encoding EGFR-mCherry and wild-type HER2-eGFP.

After transfection, the cells were allowed to grow for 24 h and then lysed. The level of EGFR-mCherry and wild-type and S310F HER2-eGFP in the cell lysate was determined by measuring the fluorescence intensity. After a calibration curve was prepared using recombinant mCherry and eGFP protein standard with a florometer, the ratios of EGFR-mCherry vs. wild-type HER2-eGFP and EGFR-mCherry vs. S310F HER2-eGFP in the cell lysates were determined to be 1:2.14 and 1:2.98, respectively. For single molecule interaction analysis, the lysate was subjected to a flow chamber coated with anti-mCherry antibody. Using the TIRF microscope, eGFP spots were identified and their intensity was determined and summed. The eGFP spot intensity summation value was increased in a dose-dependent manner as the concentration of EGFR-mCherry injected into the flow cell was increased in both the cell lysates of wild-typeHER2-eGFP and S310F HER2-eGFP transfectants (Figure 4b). Considering the ratio of wild-typeHER2-eGFP and S310F HER2-eGFP vs. EGFR-mCherry in the cell lysates of the two transfectants, the ratio of eGFP vs. mCherry in the S310F HER2-eGFP transfectants was not significantly different from that of the wild-typeHER2-eGFP transfectants (Figure 4b). Two additional independent experiments yielded similar results. Based on these observations, we concluded that S310F HER2 interacted with the EGFR and its efficiency was comparable to that of the EGFR and wild-type HER2 interactions.

## 4. Discussion

After the initiation of the Cancer Genome Anatomy Project, new genetic alterations and new driver events were found in human cancers [52]. Somatic mutations of HER2 have been identified in a wide range of solid tumors, including breast, lung, colorectal, bladder, gastric, and cervical cancers [53,54,55,56,57,58]. HER2 activating mutations have been identified mostly in the kinase domain. C515R, T526A, G776R, L755S, L869R, R897G, and P1074S mutations were reported to increase phosphorylation levels of HER2. D769H and V777L mutations increased the phosphorylation of both HER2 and EGFR [27,35]. L755S and V777L were the most studied mutations in clinical cases [59,60]. Cells with HER2 L775S mutation showed increased levels of phospho-EGFR, -HER2, and -HER3 [27]. Patients with the HER2 L755S mutation were resistant to trastuzumab and lapatinib treatment, and a non-small cell lung cancer patient with HER2 N813D mutations was resistant to afatinib [61,62]. HER2 positive metastatic colorectal cancer patients with HER2 T784G mutation did not respond to cetuximab therapy [63]. A recent study showed that cells with the HER2 L755S mutation were resistant to lapatinib [64]. HER2 activating mutations were also found on the extracellular domain. Seven mutations, including G309 and S310 mutations, were found to increase the phosphorylation level of HER2 (Table 1) [32,35]. However, the mechanism of how these extracellular mutations increase the phosphorylation of HER2 has not been clarified.

In our study, ligand-independent HER2 dimerization blocking antibody, trastuzumab, did not inhibit the phosphorylation of HER2 in 5637 cells (Figure 3a), although it bound to the S310F mutant and the wild-type HER2 (Figure 1b). Trastuzumab treatment also did not hinder the proliferation of the cells (Figure 3b). This observation proved that either S310F mutant or wild-type HER2 can be activated without forming heterodimer or homodimers among the mutant and wild-type HER2. Because 5637 cells overexpress the EGFR, we hypothesized that the S310F mutant or wild-type HER2 formed heterodimers with the EGFR. As expected, the ligand binding-blocking antibody, cetuximab, almost completely abolished the phosphorylation of not only EGFR but also HER2 (Figure 3a). As a ligand-dependent HER2 heterodimerization blocking antibody, pertuzumab did not inhibit the phosphorylation of HER2 (Figure 3a), while it bound to wild-type HER2 (Figure 1a), EGFR-mediated phosphorylation should occur on the S310F mutant HER2. In this study, we were unable to determine the level of wild-type and S310F mutant in 5637 cells at the protein level; however, the limited amount of wild-type HER2 compared to the S310F mutant might be the reason why pertuzumab is not effective. To determine whether the S310F mutant retained affinity for the EGFR, we performed single molecule interaction analyses using a TIRF microscope as described previously [49]. The S310F HER2 mutant showed an affinity equivalent to that of wild-type HER2 in the formation of EGFR/HER2 complexes (Figure 4b).

In this study, we confirmed the loss or reduction of pertuzumab reactivity to G309 and S310 mutated HER2 proteins (Figure 1a). Complementary determining region 3 (CDR3) of pertuzumab heavy chain makes hydrophobic and hydrogen bond contacts with residues K333 and H318 of HER2 [9]. Therefore, mutations on G309 and S310 would induce critical structural changes on the epitope of pertuzumab. Interestingly, pertuzumab showed reduced binding activity to G309A mutated proteins but no reactivity to the G309E mutated proteins. The loss of binding activity could be explained by the structural difference between the two mutated amino acids; alanine and glutamic acid. G309 is located close to the C299-C311 disulfide bond [65]. While both glycine and alanine have a small nonpolar side chain, the glutamic acid has a long and charged side chain, influencing the structure of the HER2 domain II by breaking the disulfide bond. This hypothesis was tested by using SDS-polyacrylamide gel electrophoresis of the mutated recombinant HER2 proteins. G309E mutated protein formed multimers in non-reducing conditions, while the wild-type and G309A mutated HER2 proteins formed monomers (Figure S1). This observation was in agreement with that of a previous study reporting that the G309E HER2 mutant formed homodimers under non-reducing conditions in SDS-polyacrylamide gel electrophoresis analysis, which could be resolved by reducing agents [20].

Our study showed that a HER2 S310F mutation induced structural changes in domain II of HER2, abolishing its reactivity to pertuzumab, but its ability to form EGFR/HER2 dimers was not affected. HER2 heterodimerization with ligand-bound HERs was through a domain II-mediated dimerization interface [66]. In contrast, HER2 heterodimerization with ligand-free HERs may be mainly involved in domain IV [67]. After activation of EGFR, trastuzumab failed to reduce the phosphorylation level of the EGFR bound to HER2 [68]. Meanwhile, pertuzumab reduced the phosphorylation level of EGFR bound to HER2 [69]. The retained reactivity of S310F HER2 mutant to EGFR observed in our study suggested the unaffected structure of HER2 domain II on the interface of HER2/EGFR heteromdimer. C213, H215, and E243 residues of HER2 that are located on the top of the dimerization arm, are reported to stabilize the heterodimer by hydrogen bonding and salt bridges, and hence HER2 S310F mutation may not have affected the heterodimerization [70].

## Figures and Tables

**Table 1:** Human epidermal growth factor receptor 2 (HER2) extracellular domain mutations reported in previous studies from patient samples.

Protein Change	Pfam Domain [8]	Tumor Type	Impact	HER2	Phosphorylation	Reference
L12R	-	Breast Cancer	ND	Negative	ND *	[35]
A20T		Lung cancer	ND	ND	ND	[24]
L49H		Lung cancer	ND	Negative	ND	[21]
L49H		Glioblastoma	ND	ND	ND	[20]
E139D	Receptor L	Breast Cancer	ND	Negative	ND	[35]
E139G		Breast Cancer	ND	Negative	ND	[35]
R157W		Bladder cancer (MPUC)	ND	Negative	ND	[32]
T216S	Furin-like	Lung cancer	ND	Negative	ND	[21]
T216S		Glioblastoma	ND	ND	High	[20]
I263T		Colorectal cancer	ND	Negative	ND	[36]
G309A		Breast Cancer	Activating	Negative	ND	[27]
G309E		Breast Cancer	Activating	Positive	ND	[24]
S310F		Gastric cancer	Activating	ND	High	[38]
S310F(2)		Breast Cancer		Negative	High	[26]
S310F		Lung cancer		Negative	High	[20]
S310F		Breast Cancer		Negative	High	[25]
S310F(2)		Colorectal cancer		Negative	High	[37]
S310F		Breast Cancer		Negative	High	[28]
S310F		Lung cancer		Positive	High	[22]
S310F		Breast Cancer		Negative	High	[29]
S310F(4)		Bladder cancer (MPUC)		Negative	High	[32]
S310F		Adnexal cancer		Negative	High	[33]
S310F		Breast Cancer		Negative	High	[29]
S310F(2)		Breast Cancer		Positive	High	[30]
S310F		Lung cancer		ND	High	[23]
S310F		Breast Cancer		Negative	High	[31]
S310F(4)		Breast Cancer		Positive	ND	[35]
S310Y		Lung cancer	Activating	ND	High	[24]
S310Y		Lung cancer		ND	High	[20]
S310Y		Colorectal cancer		Positive	High	[21]
S310Y		Bladder cancer (MPUC)		Negative	High	[20]
C311R and E321G		Lung cancer	ND	Negative	ND	[21]
C311R		Glioblastoma	ND	ND	Low	[20]
N319D		Lung cancer	ND	Negative	ND	[21]
N319D		Glioblastoma	ND	ND	ND	[20]
E321G		Glioblastoma	ND	ND	Low	[20]
D326G and C334S		Lung cancer	ND	Negative	ND	[21]
D326G		Glioblastoma	ND	ND	Low	[20]
C334S		Glioblastoma	ND	ND	ND	[20]
A466T	Receptor L	Colorectal cancer	ND	Negative	ND	[36]
A466V		Breast Cancer	Activating	Negative	High	[35]
C515R	Growth factor receptor	Breast Cancer	Activating	Negative	High	[35]
T526A		Breast Cancer	Activating	Negative	High	[35]

* ND = no data available.

**Table 2:** HER2 extracellular domain mutations reported in cell lines.

Cell Line	Protein Change	Pfam Domain [8]	Lineage	Impact	Phosphorylation
SW1271	S22N	-	Lung	ND *	ND
HEC59	R100W	Receptor L	Endometrium	ND	ND
HEC108	T166M		Endometrium	ND	ND
SBC1	Q178H		Lung	ND	ND
MOLT16	R217H	Furin-like	Haematopoietic and lymphoid tissue	ND	ND
NALM6	R226H		Haematopoietic and lymphoid tissue	ND	ND
KM12	P230L		Large intestine	ND	ND
DSH1	D277H		Urinary	ND	ND
5637	S310F		Urinary	Activating	High
DSH1			Urinary		
OACM51			Oesophagus		
HRT18	L313I		Intestine	ND	ND
NCIH2110	N319Y		Lung	ND	ND
HCC1359	T328S		Lung	ND	ND
NCIH1563	S335C		Lung	ND	ND
Jurkat	S335I	-	Haematopoietic and lymphoid tissue	ND	ND
OC314	A386T	Receptor L	Ovary	ND	ND
OC316	A386T		Ovary	ND	ND
NCIN87	F425L		Stomach	ND	ND
NCIN87	L436V		Stomach	ND	ND
SET2	T444S		Haematopoietic and lymphoid tissue	ND	ND
M059J	W452S		Central nervous system	ND	ND
ISTSL1	R499Q	-	Lung	ND	ND
SUPB8	R499W	-	Haematopoietic and lymphoid tissue	ND	ND
Jurkat	T479M	Receptor L	Haematopoietic and lymphoid tissue	ND	ND
RL952	G518V	Growth factor receptor	Endometrium	ND	ND
NCIH1793	V541M		Lung	ND	ND
NCIH740	R558M		Lung	ND	ND
Karpas45	A586G		Haematopoietic and lymphoid tissue	ND	ND

* ND = no data available.

## Supplementary Materials

The following are available online at https://www.mdpi.com/2218-273X/9/10/629/s1, **Figure S1**: SDS-polyacrylamide gel electrophoresis analysis of recombinant G309 mutant extracellular domains fused with the human Fc or Cκ domain The expression vectors encoding the fusion proteins were transfected into HEK293 cells. The recombinant proteins were purified from the culture supernatants using the affinity resin reactive to either Fc or human Cκ and subjected to 4%–12% (*w*/*v*) SDS-polyacrylamide gel electrophoresis either with or without a reducing agent. After electrophoresis, the protein bands were visualized with Coomassie Blue staining, **Figure S2**. Immunoprecipitations of HER2 in AU565 and 5637 cell lysates using trastuzumab and pertuzumab. Cell lysates were incubated with trastuzumab or pertuzumab scFv- human Cκ fusion protein followed by affinity resin reactive to human kappa light chain. After centrifugation, the amount of HER2 in the supernatant, resin, and cell lysate representing the same number of cells was determined by immunoblot analysis using anti-HER2 antibody.
