# Supplementary material for: The HER2 S310F Mutant Can Form an Active Heterodimer with the EGFR, Which Can Be Inhibited by Cetuximab but Not by Trastuzumab as well as Pertuzumab
Source: Biomolecules. 2019 Oct 19;9(10):629. doi: 10.3390/biom9100629 (PMC6843359; doi:10.3390/biom9100629)
Supplement: Supplementary file 1 [file biomolecules-09-00629-s001.pdf]

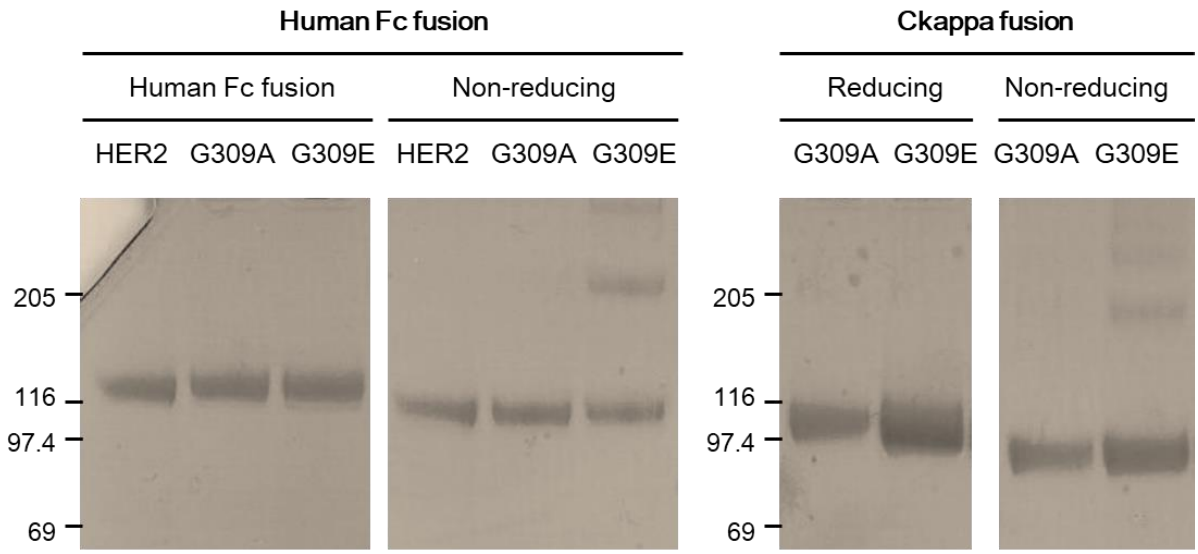

Figure S1. SDS-polyacrylamide gel electrophoresis analysis of recombinant G309 mutant extracellular domains fused with the human Fc or Cκ domain The expression vectors encoding the fusion proteins were transfected into HEK293 cells. The recombinant proteins were purified from the culture supernatants using the affinity resin reactive to either Fc or human Cκ and subjected to 4%–12% (w/v) SDS-polyacrylamide gel electrophoresis either with or without a reducing agent. After electrophoresis, the protein bands were visualized with Coomassie Blue staining.

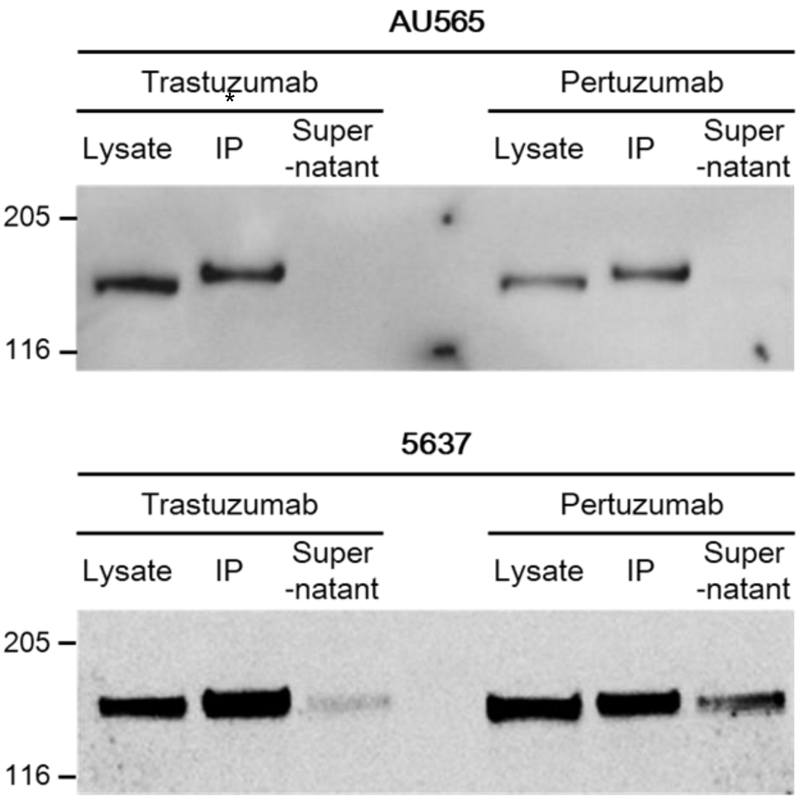

Figure S2. Immunoprecipitations of HER2 in AU565 and 5637 cell lysates using trastuzumab and pertuzumab. Cell lysates were incubated with trastuzumab or pertuzumab scFv- human Cκ fusion protein followed by affinity resin reactive to human kappa light chain. After centrifugation, the amount of HER2 in the supernatant, resin, and cell lysate representing the same number of cells was determined by immunoblot analysis using anti-HER2 antibody.

* IP = Immunoprecipitated
